# Atopic dermatitis: pathogenesis, immunology, microbiome, and salivary biomarkers

**DOI:** 10.3389/fimmu.2026.1782602

**Published:** 2026-07-29

**Authors:** Simona Abraityte, Laura Tamasauskiene, Edita Gasiuniene, Lina Jankauskaite

**Affiliations:** 1 Department of Pediatrics, Medical Academy, Lithuanian University of Health Sciences, Kaunas, Lithuania; 2 Department of Immunology and Allergology, Lithuanian University of Health Sciences, Kaunas, Lithuania; 3 Laboratory of Immunology, Department of Immunology and Allergology, Lithuanian University of Health Sciences, Kaunas, Lithuania

**Keywords:** atopic dermatitis, gut-skin axis, immune system, salivary biomarkers, skin inflammation, skin microbiome

## Abstract

Atopic dermatitis is a chronic inflammatory skin disease influenced by genetic predisposition, immune dysregulation, and environmental factors, as well as skin and gut microbiota. This review summarises the key mechanisms involved in this disease development, including disturbances in innate and adaptive immune responses, ethnic differences in immune pathways, and the contribution of microbial imbalance to skin barrier dysfunction. Special attention is given to salivary biomarkers—such as cortisol, melatonin, alpha-amylase, chromogranin A, C-reactive protein, and immunoglobulin A—which offer a non-invasive method for monitoring stress, sleep quality, and disease severity in patients with atopic dermatitis. Understanding the complexity of these factors may support earlier diagnosis and different treatment strategies and improve long-term disease management in a better way.

## Highlights

- This review highlights how the interaction between genetic factors, immune dysregulation, skin and gut microbial imbalances and stress-related salivary biomarkers collectively contributes to the development, progression and exacerbation or remission of atopic dermatitis.

## Introduction

Atopic dermatitis (AD) also known as eczema is a chronic, relapsing inflammatory skin disease whose prevalence has increased two to three times in developed countries over the past decades. Currently, it affects approximately 15%–20% of children and 1%–3% of adults worldwide (1–4). The condition usually appears in infancy, with 50%–60% of cases developing in the first year and 90% of cases by the age of 5. Most of AD cases start in childhood. Three main symptoms of AD are xerosis (dry skin), skin lesions, and intense itching. Skin lesions can present as plaques, eczema, and oozing lesions, which vary by age. Chronic inflammation and repeated scratching often lead to skin thickening—a condition known as lichenification. Persistent itching is the main symptom that disrupts the quality of daily life and sleep (5). People with AD are at a higher risk of other allergic diseases such as asthma, food allergies, and hay fever. Additionally, they are more susceptible to bacterial, viral, and fungal skin infections due to a compromised skin barrier. Environmental factors, including exposure to allergens, pollution, infections, antibiotic use, breastfeeding duration, diet, and harsh chemicals, can exacerbate AD symptoms. Moreover, genetic predisposition also plays a significant role with mutations in the Filaggrin (FLG) gene being most strongly associated with AD (also it is associated with *Ichthyosis vulgaris*). This disease is multifactorial, which means that it arises from a combination of environmental, genetic, immunological, and microbiome-related factors (1, 6). In this article, we will discuss atopic dermatitis pathogenesis, immunology, microbiome, and salivary biomarkers. Despite advances in atopic dermatitis research, effective treatment still remains limited, leaving many patients struggling with persistent symptoms that significantly impact their quality of life. Given these challenges, salivary biomarkers have emerged as a promising tool for monitoring disease progression and understanding the interplay between levels of stress and severity of eczema. Salivary biomarkers are a non-invasive alternative to traditional blood tests, making them particularly suitable for children and individuals who require frequent monitoring. Salivary biomarkers could provide valuable insights into acute and chronic stress, which are known to exacerbate AD symptoms. Stress-related fluctuations in cortisol, melatonin, alpha-amylase, chromogranin A, CRP, and immunoglobulin A (IgA) levels can be tracked dynamically, helping clinicians assess the relationship between psychological stress, disease severity, exacerbation, and overall well-being (7, 8). Melatonin, another salivary biomarker, is thought to play an important role in atopic dermatitis. Since severe atopic eczema often disrupts sleep quality, reduced concentrations of melatonin may be detected in the body (9).

## Methods

Search strategy – on Medline via PubMed restricted mostly to paediatric age by using medical terms referring to atopic dermatitis (“atopic dermatitis,” “salivary biomarkers,” “skin–gut axis,” “pathogenesis, “immune system,” “skin microbiota,” “gut microbiota,” “itch mediators”). Some studies were excluded if the information was not specific to atopic dermatitis.

## Immune system dysregulation in atopic dermatitis (innate immunity)

The immune response consists of two main components: the innate response and the adaptive response. The skin’s immune defence system in AD patients includes the epidermis, immune cells, and cytokine receptors that detect harmful substances like pattern recognition receptors (PRRs), antimicrobial proteins, and skin microbiome. PRRs help the immune system recognise harmful bacteria and viruses (1, 2, 10). Due to barrier impairment in atopic dermatitis cases—keratinocytes and dendritic cells are exposed to pathogen-associated molecular patterns (PAMPs) and endogenous damage-associated molecular patterns (DAMPs) which are sensitised due to Toll-like receptors (TLRs) (11, 12). Keratinocytes, expressing multiple TLRs, become hyperresponsive and release alarmins such as TSLP, IL-33, and IL-25, driving ILC2 and Th2-skewed inflammation. TLR2 and TLR4 activation by *Staphylococcus aureus* components amplifies NF-κB-mediated cytokine production, whereas viral nucleic acids activate endosomal TLR3/7/8/9, further enhancing inflammation (13). Barrier-derived DAMPs (e.g., nucleic acids and ATP) sustain sterile inflammation even in the absence of infection. Together, these innate immune pathways form the mechanistic basis of chronic type-2 inflammation in AD (1, 2, 5, 10–16).

## Immune system dysregulation in atopic dermatitis (adaptive immunity)

The skin’s immune cells support the body’s defence system by triggering an immune response that produces special molecules antimicrobial peptides (AMPs) which are designed to kill or block harmful microbes. Changes in the production or amount of antimicrobial peptides (AMPs) are linked to atopic dermatitis. AMPs from human skin commensals protect against *S. aureus* (2, 6, 17). Innate lymphoid cells (ILCs) are the group of immune cells that play a role not only in immune defence but also in remodelling tissues. These cells function like T cells but do not have specific antigen receptors. They help regulate immune responses by producing cytokines and interacting with other cells in the body. ILCs are located in the upper skin layers and are classified into three main types based on the cytokines they produce and their roles in immunity: ILC1, ILC2, and ILC3. ILC2 cells are a type of ILC which is known for releasing pro-allergic cytokines like IL-4, IL-5, IL-9, and IL-13, which are linked to allergic diseases. This suggests that ILC2 plays a role in triggering Th2-type immune responses, which are involved in allergies and atopic dermatitis as well. In atopic dermatitis, CD4 lymphocytes tend to develop into Th2 cells, which produce IL-4, IL-5, and IL-13 (10, 15, 18). These cytokines stimulate IgE antibodies and eosinophils by increasing allergic inflammation. The Th1 response, characterised by IL-2, IL-12, TNFα, and INF-γ, becomes more active, contributing to long-term inflammation. Other immune cells, such as Th17 and Th22, release cytokines like IL-17, IL-19, and IL-22, which may also contribute to skin damage. Recent studies suggest that IL-36 family cytokines (IL-36α, IL-36β, IL-36γ, IL-36Ra, and IL-38) could be involved in atopic dermatitis pathogenesis, adding another layer of complexity to the disease. In acute lesions, there is an increase in Th2 and Th22 cytokines, with some involvement of Th17. As the disease progresses (for example chronic lesions), the Th1 response increases and Th2 and Th22 responses become stronger. **Table 1** shows immunity dysregulation in role of AD. However, more recent studies show that Th2 cytokines are overexpressed through both phases of the disease, so pathogenesis in not completely clear (1, 2, 6, 10, 15, 34).

**Table 1:** Innate, adaptive immunity components and functions in the role of AD.

Component	Function	Impact on AD
Innate immunity		
Pattern recognition receptors (PRRs)	Recognises pathogens via PAMPs	Dysregulation affects skin barrier integrity
Toll-like receptors (TLRs)	First discovered PRRs, trigger inflammatory response	Mutations can impair microbial recognition and increase susceptibility to infections
NOD-like receptors (NLRs)	Involved in inflammatory signalling pathways	Over-activation leads to chronic inflammation in AD
C-type lectin receptors (CLRs)	Recognises fungal and bacterial pathogens	Dysfunction may contribute to microbial imbalance in AD
Antimicrobial peptides (AMPs)	Naturally produced molecules that fight pathogens	Reduced AMP levels linked to *Staphylococcus aureus* colonization
Innate lymphoid cells (ILCs)	Regulate immune defence and tissue remodelling	ILC2 releases cytokines (IL-4, IL-5, IL-9, IL-13) contributing to allergic inflammation
Adaptive immunity		
T-helper (Th) cells	Coordinate immune responses	Th2 dominance leads to allergic inflammation, whilst Th1 contributes to chronic AD
Th2 cells	Produce IL-4, IL-5, and IL-13 (pro-allergic cytokines)	Excessive activation fuels IgE-mediated allergic responses
Th1 cells	Produces IL-2, IL-12, TNFα, and INF-γ	Increases during chronic inflammation in AD
Th17 cells	Releases IL-17, IL-19, and IL-22	Can exacerbate skin damage, varies by ethnicity
Th22 cells	Secretes IL-22, affecting keratinocyte proliferation	Involved in skin barrier dysfunction
IgE and eosinophils	Mediate allergic immune responses	High IgE levels intensify inflammation via FcϵRI on skin cells
Ethic variability		
Population differences	Variations in Th responses between ethnic groups	Th2/Th22 dominant in Europeans/Americans, Th17 stronger in Asians

## Immune system dysregulation in atopic dermatitis due to ethnicity and allergic history

The immune responses in AD could also differ by ethnicity. In European and American populations, Th2 and Th22 responses dominate with lower Th1 and Th17 responses. However, Asian patients (e.g., from Japan) show stronger Th17 responses and weaker Th1 responses. Chinese patients also exhibit Th2 activation but with an increased Th17/IL-23 response. In African Americans, Th2 and Th22 responses are more pronounced, whilst Th1 and Th17 responses are weaker (1, 6). Patients with AD often have higher levels of IgE and eosinophils along with a positive family history of allergic diseases. External irritants and damage to the skin barrier could trigger a Th2 response leading to the production of IgE antibodies. These antibodies bind to FcϵRI on skin cells, which intensifies the immune response and inflammation in the skin. However, there is an ongoing debate about whether autoimmunity plays a significant role in AD with arguments against this theory based on the lack of clear links between IgE levels and disease severity and mixed results from eliminating allergens (1, 2, 10, 19–21). Moreover, patients with severe Th2-dominant disease or long-standing allergic comorbidities may experience more chronic itch, sleep disruption, and psychological stress, potentially leading to higher cortisol or α-amylase levels and reduced melatonin or IgA concentrations. Ethnic differences in circadian biology, stress perception, microbiome composition, and immune activation may further contribute to inter-patient variability in salivary biomarker patterns. Although these associations remain exploratory, current evidence is insufficient to draw firm conclusions or to identify clear differences between immunological endotypes and salivary biomarker profiles. Existing studies are limited in number, heterogeneous in methodology, and often underpowered, making it difficult to determine whether distinct biomarker patterns truly reflect ethnicity-related immune variation, allergic history, or disease severity. More robust, well-designed studies are needed before meaningful clinical distinctions can be established (2, 10, 19, 20).

## Microbiota and skin (skin–gut axis) in atopic dermatitis

**Figure 1:**
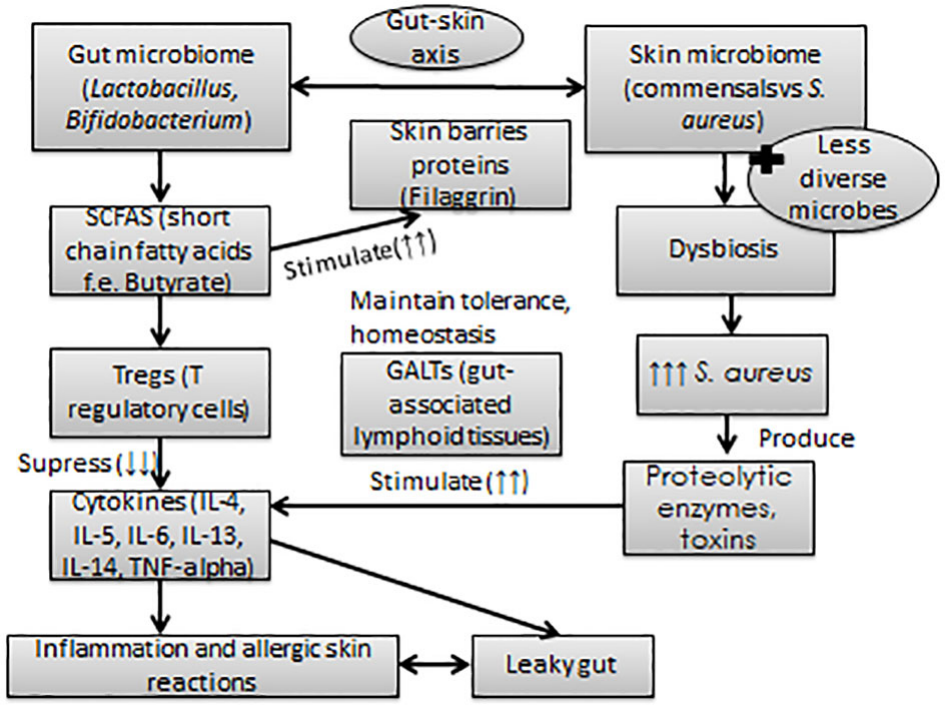
Gut–skin axis.

Atopic dermatitis can be influenced by microbial imbalances as well. This connection between the gut microbiome and skin health is known as the “gut–skin axis,” and it plays a significant role in allergic diseases like AD. The bacteria and other microorganisms living on the skin and in the gut play a key role in AD development. Changes in the balance and variety of these microorganisms can influence how AD starts and progresses—it is known as dysbiosis, and it is associated with skin inflammatory responses and skin barrier dysfunction. For example, having less diverse microbes is linked to more severe symptoms, especially in areas of the skin affected by AD (22–24). The skin is home to large numbers of commensal microbes. The skin microbiome, which includes bacteria and fungi, varies based on factors such as age, gender, and environmental influences. Changes in the skin microbiome, such as an overgrowth of harmful microbes like *Staphylococcus aureus*, are commonly found in AD patients. This bacterium produces substances that trigger inflammation and worsen skin symptoms by stimulating immune responses, such as Th2 cytokines: *S. aureus* produces toxins and proteolytic enzymes that degrade the epidermal barrier and stimulate Th2 cytokines (IL-4, IL-5, and IL-13), promoting inflammation and allergic skin reactions. The imbalance in the skin microbiome can lead to inflammation. However, it is still not clear if *S. aureus* is the cause of the disease or consequence. Moreover, there are also commensal bacteria in the gut microbiome, which play an important role in the gut and skin health in general. For example, beneficial bacteria such as *Lactobacillus* and *Bifidobacterium* contribute to immune tolerance and the production of short-chain fatty acids (SCFAs), which promote anti-inflammatory pathways. Those SCFAs can help regulate immune response by activating Tregs (T regulatory cells). Tregs can suppress Th2-driven inflammation, which is one of the main pathogeneses in AD. These cells can inhibit pro-inflammatory cytokines such as IL-6, IL-17, and TNF-alpha. One of the SFAs called butyrate can stimulate filaggrin and loricrin (proteins, which are responsible for skin barrier function) expression (6, 22, 23, 25). It could reduce skin permeability, and it will not let trigger an immune response to allergens and irritants (24). However, in AD patients, the SCFA levels are reduced due to uncontrolled inflammation (22–24). Gut-associated lymphoid tissues (GALTs) help shape immune responses and maintain tolerance to harmless substances; they play an important role in immune homeostasis (23, 24). In AD patients, homeostasis is disrupted leading to hyperreactive immunity. However, some studies suggest that patients with AD may exhibit “leaky gut” (intestinal permeability) (26). **Figure 1** visually illustrates the mechanism described above. Such permeability changes could allow microbial or dietary antigens to enter the circulation and contribute to systemic inflammation with potential effects on the skin. The gut microbiome plays an important role in regulating immune responses, and disturbances in this ecosystem have been associated with AD and other allergic diseases. Therefore, maintaining the health of both the gut and skin microbiomes may be important for supporting immune balance and reducing the risk of inflammatory conditions such as atopic dermatitis (6, 22, 23, 25).

## Itch in atopic dermatitis

The itch (pruritus) in atopic dermatitis (AD) is one of the main symptoms and is also caused by multiple interacting pathways. First of all, histamine is a biogenic amine (2-[4-imidazolyl]-ethylamine) and is involved in pruritus. Mast cells, basophils, and eosinophils are the main cells with sources of histamine. However, other cells, including T cells, dendritic cells, and even keratinocytes have been shown to produce histamine in response to stimulation. While histamine is a classic itch mediator and exerts through G protein-coupled receptors (H1 and H4 receptors), it also plays a minor role in AD itch compared to urticarial itch. We can even observe this in clinical practice, where antihistamines are less effective in atopic dermatitis compared to treatment of urticaria. The aetiology of itch in atopic dermatitis (AD) is multifactorial with histaminergic itch being only one of several contributing pathways (2). Moreover, neuropeptides (e.g., substance P and calcitonin gene-related peptide (CGRP)) play an important role in the atopic dermatitis itch pathway. Neuropeptides like substance P CGRP are involved in chronic itch through neuropathic itch pathways (4). Skin cells, such as keratinocytes and mast cells, can release nerve growth factor (NGF), which increases the number of nerve fibres. This, in turn, makes the inflammatory response stronger. Prolonged exposure of the skin to external triggers can lead to inflammation, resulting in skin damage. Other substances that play a huge role in pruritus of AD patients are cytokines. They are usually produced by Th2 cells, and the main ones are IL-31, TSLP, IL-4, and IL-13. IL-31 is highly associated with pruritus; it is even called “the itch cytokine” (5, 14, 27). IL-31 binds to the IL-31 receptor complex, which is present on sensory neurons in the skin. This binding activates the neurons, leading to the sensation of itching. Elevated levels of IL-31 are strongly correlated with the severity of itch in individuals with AD (3, 4). Another cytokine is TSLP (thymic stromal lymphopoietin), and this cytokine is released by keratinocytes when the skin is damaged. It activates sensory neurons and helps drive Th2 inflammation, further contributing to the itch of AD (5, 14, 27). There are other cytokines, like IL-4, that play an important role in the itch pathways of atopic dermatitis. IL-4 impairs the skin barrier, promotes histamine release, activates skin inflammation, and indirectly contributes to chronic itch (28). Proteases derived from mast cells, keratinocytes, and allergens contribute to pruritus by activating protease-activated receptors (PARs) on sensory nerves. For example, tryptase, a mast cell protease, activates PAR-2, promoting nerve sensitization and itch. Kallikreins, epidermal serine proteases, disrupt the skin barrier and stimulate sensory nerves. Lipid mediators derived from arachidonic acid also play a role in neuro-inflammation and the itch. Prostaglandins (PGE2 and PGD2) sensitise sensory neurons and enhance the itch response, whilst leukotrienes (LTB4) promote inflammation and may amplify sensory nerve signalling in cells. Lipoxins, which are anti-inflammatory lipid mediators, have diminished function in atopic dermatitis (29). To sum up, the itch in atopic dermatitis arises from a multifactorial interplay of immune-derived cytokines, neuropeptides, proteases, and lipid mediators, which sensitise peripheral sensory nerves and drive chronic inflammation. Histamine, whilst a classical pruritogen, plays a relatively minor role in AD compared to other mediators (3, 5, 14, 27–29). **Table 2** shows itch mechanism of AD.

**Table 2:** Itch mediators in atopic dermatitis.

Category	Mediators/factors	Source/action	Role in AD itch
Histamine	Histamine	Produced by mast cells, basophils, eosinophils, T cells, dendritic cells, keratinocytes; acts via H1 and H4 receptors	Plays a minor role in AD itch; more prominent in urticaria
Neuropeptides	Substance P, CGRP	Involved in chronic/neuropathic itch pathways; stimulate sensory nerves	Contribute to neurogenic inflammation and chronic itch
Nerve growth factor (NGF)	NGF	Released by keratinocytes and mast cells; increases nerve fibre density	Enhances sensitivity of nerves, amplifies itch response
Cytokines (Th2-derived)	IL-31	Binds to IL-31 receptor on sensory neurons; called “itch cytokine”	Strongly correlated with itch severity in AD
Cytokines (Th2-derived)	TSLP	Released by damaged keratinocytes; activates sensory neurons and Th2 cells	Drives inflammation and contributes to itch
Cytokines (Th2-derived)	IL-4, IL-13	Promote histamine release, impair skin barrier, activate sensory nerves	Indirectly contribute to chronic itch
Proteases	Tryptase (mast cell)	Activates PAR-2 on sensory nerves	Promotes nerve sensitization and itch
Proteases	Kallikreins (keratinocytes)	Disrupt skin barrier, stimulate nerves	Contribute to itch and barrier dysfunction
Lipid mediators	Prostaglandins (PGE2, PGD2)	Sensitise sensory neurons	Enhance itch response
Lipid mediators	Leukotrienes (LTB4)	Promote inflammation, amplify nerve signalling	May exacerbate pruritus
Lipid mediators	Lipoxins	Anti-inflammatory lipid mediators (diminished in AD)	Reduced lipoxin function may allow more inflammation and itch

## Salivary biomarkers in atopic dermatitis

Saliva in the human’s body is produced by three main glands—the parotid, submandibular, and sublingual glands—99% of saliva contains water, and less percentage of saliva contains many other substances such as electrolytes, proteins, lipids, and enzymes, bacteria, epithelial cells, gingival fluid, and food debris (30). Moreover, it contains stress-related biomarkers (7, 27, 30–32). The autonomic nervous system controls saliva production: the parasympathetic system mainly regulates fluids and electrolytes, whilst the sympathetic system stimulates the release of proteins (31). Salivary biomarkers can provide valuable insights into acute and chronic stress, which are known to exacerbate AD symptoms (7, 27, 30–32). It is possible to measure such substances like cortisol, chromogranin A, alpha-amylase, melatonin, and IgA in saliva (7). Stress-related fluctuations in cortisol, melatonin, alpha-amylase, and immunoglobulin A (IgA) levels can be tracked dynamically, helping clinicians assess the relationship between psychological stress, disease severity, and overall well-being (life quality index). Patients with AD often experience sleep disturbances, which further intensify stress levels and create a vicious cycle of worsening symptoms and reduced quality of life. By analysing salivary biomarkers, clinicians could explore the connections between stress, sleep quality, and immune responses, potentially leading to more personalised treatment strategies and patient care. Measuring of salivary biomarkers is an especially suitable method for paediatric patients, who are often fearful of needles and invasive procedures. There are some salivary biomarkers which could give some meaning in AD patient clinical data (7, 27, 32). **Table 3** shows salivary biomarkers control and functions in AD patients:

1. Cortisol—this biomarker is produced by the hypothalamic–pituitary–adrenal (HPA) axis; it is an acute stress hormone and elevates to provide energy. Patients with AD could have higher cortisol levels in saliva due to stress because of chronic itch, skin condition, and disease-related psychological burden. It can further impair epidermal barrier repair, alter keratinocyte differentiation, and modulate Th2-skewed inflammation (7, 31, 32). One study showed that cortisol levels were significantly higher in patients with atopic dermatitis compared to healthy controls and showed a positive correlation with disease severity (measured by the SCORAD index), whilst no significant association was found with conventional inflammatory biomarkers or quality-of-life measures. These findings suggest that salivary cortisol may serve as a potential non-invasive biomarker of disease activity and physiological stress in atopic dermatitis (32).
2. α-Amylase and chromogranin A—markers of the sympathetic–adrenal–medullary (SAM) system activation could be elevated in individuals experiencing chronic stress, including adolescents with AD. Chromogranin A is an acidic glycoprotein released from chromaffin granules of the adrenal medulla and sympathetic nerve endings. α-Amylase in an enzyme secreted by the salivary glands under adrenergic stimulation (7).
3. Melatonin—circadian hormone. Melatonin is a circadian neurohormone primarily produced by the pineal gland, regulating sleep–wake cycles and circadian rhythm, and exerting antioxidant, anti-inflammatory, and immunomodulatory effects. It could be decreased due to chronic stress because of nocturnal pruritus in children with atopic dermatitis. Melatonin may influence AD pathophysiology by reducing oxidative stress, supporting epidermal barrier integrity, and modulating Th2-driven inflammation, including reduction of total IgE and IL-4 levels (7, 9).
4. IgA—mucous immunoglobulin; it could play an important role in defending against infections. Low IgA levels could be linked to more severe AD cases and may indicate higher stress levels (7).

**Table 3:** Salivary biomarkers.

Biomarker	Origin/control	Function	Relevance to AD
Cortisol	HPA axis (acute stress hormone)	Provides energy during stress	Elevated in AD due to chronic stress from itching and skin lesions
Chromogranin A	Sympathetic nervous system	Indicates stress and sleep disturbances	Higher levels in severe AD; reflects increased stress
Alpha-amylase	Sympathetic nervous system	Enzyme responsive to psychological stress	Elevated in chronic stress, common in teenage AD patients
Melatonin	Pineal gland, regulated by circadian rhythm	Regulates sleep	Often decreased in AD due to poor sleep quality caused by itching
Immunoglobulin A (IgA)	Mucosal immune system	Defends against infections	Lower levels in severe AD; linked to higher stress

However, substantial inter-patient variability limits the interpretation of individual salivary biomarkers. Salivary biomarkers’ concentrations are influenced by circadian rhythm, age, pubertal stage, comorbidities, medication use, oral health, and individual stress reactivity (7). There is limited data about salivary biomarkers in AD patients—interpretation of single biomarkers in atopic dermatitis stage, severity, and other important information is not quite clear. It could show multidimensional interactions between stress, immune activation, and disease severity (7). Further research is needed to validate their diagnostic and prognostic value, ensuring they can be effectively integrated into care (7, 27), although their non-invasive nature and ability to track disease dynamics make them a promising tool for improving patient outcomes and quality of life in the future. It could be a great tool for clinical practice in future perspective of monitoring AD patients and trying to approach more individualised medicine in a paediatric population. Salivary biomarkers represent a novel diagnostic tool for assessing disease severity and control in atopic dermatitis (AD) patients with some potential for future integration into clinical practice (7, 27, 30–33). However, current evidence is limited, preventing firm conclusions about their diagnostic value or their ability to distinguish between AD endotypes, severity levels, or treatment responses. More robust, longitudinal studies are required to determine whether salivary biomarkers can reliably reflect disease dynamics or guide individualised management strategies (7, 27, 30–33). **Table 4** shows atopic dermatitis pathogenesis and associated salivary biomarkers.

**Table 4:** Atopic dermatitis pathogenesis and associated salivary biomarkers.

Pathophysiology	Mechanisms	Clinic	Salivary biomarker/s
Skin barrier dysfunction	Filaggrin deficiency, ↑ TEWL, microfissures, allergen penetration	Barrier fragility, microbial, allergen entry	–
Innate immunity	TSLP, IL-33, IL-25 release; activation of ILC2, mast cells, dendritic cells; TLR2/4/7/9 signalling; NF-κB → Th2 inflammation	Eczema, erythema, inflammation	Cortisol ↑
Microbiome dysbiosis	*S. aureus* overgrowth, ↓ microbial diversity, protease activity, toxin production	Barrier damage, inflammation, pruritus	α-Amylase ↑
Melatonin	IL-31, TSLP, neuroimmune sensitization, nerve fibre hyperreactivity	Itch-scratch cycle, insomnia	Melatonin ↓
Psychological stress	Chronic itch → emotional burden; visible lesions → psychosocial stress; HPA + SAM activation	Sleep disturbance, lower quality of life, exacerbation of AD	Cortisol ↑, α-amylase ↑, Chromogranin A ↑
Chronic stress	Impaired mucosal immunity, prolonged HPA/SAM activation	Exacerbation of AD, secondary infections	IgA ↓

## From immune dysregulation to stress and salivary biomarkers

Immune dysregulation in AD patients mentioned before further alters the skin microbiome, promoting *Staphylococcus aureus* overgrowth and reducing microbial diversity. Microbial imbalance reinforces inflammation through additional TLR activation and contributes to pruritus via protease activity, barrier damage, and neuroimmune signalling. Persistent itch leads to scratching, which exacerbates barrier breakdown and perpetuates the inflammatory cycle (4, 6, 14, 15, 22, 28). Chronic pruritus and visible skin lesions significantly increase psychological stress, activating both the hypothalamic–pituitary–adrenal (HPA) axis and the sympathetic–adrenal–medullary (SAM) system. These neuroendocrine responses could influence salivary biomarkers such as cortisol, which rises with HPA activation. Alpha-amylase and chromogranin A reflect sympathetic activity, melatonin decreases due to sleep disruption caused by nocturnal itch, and IgA may decline under chronic stress. These biomarker fluctuations could mirror the physiological burden of AD and may correlate with disease severity, sleep quality, and stress-related exacerbations [11, 22. 24. 26, 32]. The barrier dysfunction, immune activation, microbiome imbalance, and neuroendocrine stress responses form a tightly interconnected pathophysiological network. Salivary biomarkers represent downstream indicators of this network, capturing the cumulative impact of inflammation, pruritus, sleep disturbance, and psychological stress. Although evidence remains preliminary, integrating these biomarkers with immunological and microbial profiles may help identify patient-specific stress–immune signatures and support more individualised approaches to AD management (7, 8, 31, 32).

## Conclusion

In conclusion, atopic dermatitis (AD) is a multifactorial disease influenced by genetic, environmental, immune, and microbiome-related factors. It significantly impacts the quality of life due to chronic inflammation, persistent itching, and skin barrier dysfunction. The dysregulation of both innate and adaptive immune responses—particularly involving Th2, Th22, and Th17 pathways—plays an important role in the disease’s progression. The skin and gut microbiomes further contribute to atopic dermatitis severity with homeostasis imbalances, especially the overgrowth of *Staphylococcus aureus* by worsening symptoms. The persistent itch has complexed pathways, including cytokines like IL-31, neuropeptides, and proteases, rather than histamine alone. Additionally, salivary biomarkers show promise for non-invasive monitoring of stress and disease severity, offering potential for more personalised treatment strategies. Understanding these mechanisms of this common disease is a key to developing more effective targeted therapies for managing AD symptoms.
